# *MET* FISH-positive status predicts short progression-free survival and overall survival after gefitinib treatment in lung adenocarcinoma with *EGFR* mutation

**DOI:** 10.1186/s12885-015-1019-1

**Published:** 2015-02-06

**Authors:** Rintaro Noro, Masahiro Seike, Fenfei Zou, Chie Soeno, Kuniko Matsuda, Teppei Sugano, Nobuhiko Nishijima, Masaru Matsumoto, Kazuhiro Kitamura, Seiji Kosaihira, Yuji Minegishi, Akinobu Yoshimura, Kaoru Kubota, Akihiko Gemma

**Affiliations:** Department of Pulmonary Medicine and Oncology, Graduate School of Medicine, Nippon Medical School, Tokyo, Japan; Department of Clinical Oncology, Tokyo Medical University Hospital, Tokyo, Japan

**Keywords:** *MET*, Lung cancer, Fluorescence *in situ* hybridization, Gefitinib, EGFR mutation

## Abstract

**Background:**

Lung adenocarcinoma patients with *EGFR* gene mutations have shown a dramatic response to gefitinib. However, drug resistance eventually emerges which limits the mean duration of response. With that in view, we examined the correlations between *MET* gene status as assessed by fluorescence *in situ* hybridization (FISH) with overall survival (OS) and progression-free survival (PFS) in adenocarcinoma patients with *EGFR* gene mutations who had received gefitinib therapy.

**Methods:**

We evaluated 35 lung cancer samples with *EGFR* mutation from adenocarcinoma patients who had received gefitinib. Gene copy numbers (GCNs) and amplification of *MET* gene before gefitinib therapy was examined by FISH. MET protein expression was also evaluated by immunohistochemistry (IHC).

**Results:**

FISH assessment showed that of the 35 adenocarcinoma samples, 10 patients (29%) exhibited high polysomy (5 copies≦mean MET per cell) and 1 patient (3%) exhibited amplification (2≦*MET gene* (red)/*CEP7q* (green) per cell). IHC evaluation of MET protein expression could not confirm *MET* high polysomy status. The Eleven patients with *MET* FISH positivity had significantly shorter progression-free survival (PFS) and overall survival (OS) than the 24 patients who were *MET* FISH-negative (PFS: p = 0.001 and OS: p = 0.03). Median PFS and OS with MET FISH-positivity were 7.6 months and 16.8 months, respectively, whereas PFS and OS with MET FISH-negativity were 15.9 months and 33.0 months, respectively. Univariate analysis revealed that *MET* FISH-positivity was the most significant independent factor associated with a high risk of progression and death (hazard ratio, 3.83 (p = 0.0008) and 2.25 (p = 0.03), respectively).

**Conclusions:**

Using FISH analysis to detect high polysomy and amplification of *MET* gene may be useful in predicting shortened PFS and OS after Gefitinib treatment in lung adenocarcinoma. The correlation between *MET* gene status and clinical outcomes for EGFR-TKI should be further evaluated using large scale samples.

## Background

Activating mutations of the epidermal growth factor receptor (*EGFR*) gene, including the deletion mutations around nucleotide residue 746–750 in exon 19 (exon 19 deletion) and also substitution of leucine with arginine at codon 858 in exon 21 (exon 21 L858R), are correlated with sensitivity to EGFR-tyrosine kinase inhibitors (EGFR-TKIs) [1,2]. Approximately 80% of activating *EGFR* mutant cases shows a dramatic response to EGFR-TKIs [3]. In recent phase III trials of the EGFR-TKI, gefitinib, demonstrated a significant superiority on progression-free survival (PFS) over standard chemotherapies as the first-line treatment for *EGFR*-mutated advanced non–small cell lung cancer (NSCLC) [4,5]. However, despite of the existence of *EGFR* gene activating mutations, the mean duration of many patients’ successful response to gefitinib is shortened as they acquire drug resistance. Mechanisms of acquired resistance to EGFR-TKI have recently been found, such as T790M secondary mutation and *MET* amplification [6,7].

*MET* amplification is recognized as one of the acquired mechanisms of resistance to EGFR-TKIs [7]. Although MET activation is relatively rare in patients with *EGFR* mutations before EGFR-TKI treatments, *MET* gene amplification based on clonal selection later appears at the relapse stage [8]. A recent report demonstrated that *MET* gene activation as assessed by fluorescence *in situ* hybridization (FISH) analysis contributed to poor prognosis in NSCLC patients who received surgical treatments [9]. Though it is very difficult to predict resistance to EGFR-TKI before EGFR-TKI therapy and then PFS and OS after EGFR-TKI treatment because of *MET* gene activation, there may still be a substantial clinical benefit for assessing MET FISH status in NSCLC patients with *EGFR* gene mutations especially before initiation of EGFR-TKI therapy.

In this study, we investigated if *MET* gene copy number status as assessed by FISH could predict the clinical outcome for EGFR-TKI in *EGFR*-mutated lung adenocarcinoma patients.

## Methods

### Patients and clinical features

Thirty-five tumor specimens with *EGFR* gene mutations were obtained from 35 lung adenocarcinoma patients, all of whom had received gefitinib and provided written informed consent, at Nippon Medical School Hospital between 2008 and 2010 (Table 1). Tumor samples were obtained by resections, aspirated pleural/cardiac effusion, and transbronchial lung biopsies. Patients’ characteristics are shown in Table 1. Seventeen patients had relapses despite complete tumor resection. Eighteen patients had stage III and IV cancers according to the World Health Organization TNM staging 7^th^ Edition. Response to gefitinib was evaluated by Response Evaluation Criteria in Solid Tumors (RECIST) version 1.0. This study was approved by Nippon Medical School Hospital’s Institutional Review Board. Every patient has a signature of informed consent.

**Table 1:** **EGFR and MET gene status of 35 lung adenocarcinoma cases**

						**Before Gefitinib treatments**			**At Gefitinib treatment failure**	
**Case No** ^**b**^	**Smoking**	**Staging**	**Gefitinib response**	**EGFR mutation type**	**MET FISH status**	**MET IHC**	**EGFR mutation type**	**MET FISH status**	**PFS(Months)**	**OS(Months)**
1	-	IV	PR	*Ex19 deletion*	**++**	**+**	*Ex19 deletion*	**++**	**7.3**	15
2	-	IV	PR	*Ex19 deletion*	**+**		Not detected	**+**	**7.6**	**11.9**
3	+	Relapse	PR	*Ex19 deletion*	**+**		*Ex19 deletion/T790M*	**+**	**18.5**	**34.4**
4	-	Relapse	PR	*Ex19 deletion*	**+**	**-**			**16.6**	**20.9**
5	+	IIIA	PR	*Ex19 deletion*	**+**				**8.5**	20
6	-	Relapse	PR	*Ex19 deletion*	**+**				1.8	1.8
7	-	IV	PD	*L858R*	**+**		*L858R*	**+**	**13.6**	**15**
8	+	Relapse	PD	*L858R*	**+**	**+**			**2.7**	**8.8**
9	-	Relapse	CR	*L858R*	**+**	**-**			**13**	28.4
10	-	Relapse	PR	*L858R*	**+**	**-**			7.6	11.2
11	+	IV	PR	*L858R*	**+**				**5.7**	7.3
12	+	IV	PR	*Ex19 deletion*	**-**		*Ex19 deletion*	**-**	**13.9**	21
13	+	IV	PR	*Ex19 deletion*	**-**		*Ex19 deletion*	**-**	**10.4**	36.4
14	-	IV	PR	*Ex19 deletion*	**-**		*Ex19 deletion*	**-**	**24.7**	**31.1**
15	-	Relapse	PR	*Ex19 deletion*	**-**	-	*Ex19 deletion/T790M*	-	**28.4**	33.8
16	-	Relapse	PR	*Ex19 deletion*	**-**	-			**11.1**	**21**
17	+	Relapse	PR	*Ex19 deletion*	**-**	-			**24.4**	24.4
18	-	IIIA	PR	*Ex19 deletion*	**-**				6.5	14.9
19	-	IIIB	PR	*Ex19 deletion*	**-**				29.8	38.2
20	+	IIIB	PR	*Ex19 deletion*	**-**				**41.5**	**48.6**
21	-	IV	PR	*Ex19 deletion*	**-**				13.2	26.7
22	+	IV	PR	*Ex19 deletion*	**-**				17.1	17.1
23	-	IV	PR	*Ex19 deletion*	**-**				**13.3**	**19.2**
24	-	IV	PR	*Ex19 deletion*	**-**				21.8	25.5
25	-	IV	PR	*Ex19 deletion*	**-**				**15.2**	31.6
26	-	IV	SD	*Ex19 deletion*	**-**				**3.4**	**26.8**
27	+	Relapse	PR	*Ex19 deletion*	**-**				**18.7**	50.2
28	+	Relapse	PR	*Ex19 deletion*	**-**				15.9	20.2
29	-	Relapse	PR	*Ex19 deletion*	**-**				**12.1**	**15**
30	+	Relapse	SD	*Ex19 deletion*	**-**				**11.1**	**13.6**
31	-	Relapse	PR	*L858R*	**-**		*L858R*	-	**58.7**	123.5
32	-	IV	PR	*L858R*	**-**	**-**			4.5	4.5
33	+	Relapse	PR	*L858R*	**-**				**15.5**	**16.7**
34	+	Relapse	PR	*L858R*	**-**				13.5	13.5
35	-	Relapse	PR	*L858R*	**-**				7.1	7.1

MET FISH status; ++Amplification, +High polysomy.

^b^Patient identifiers have been removed and relabeled.

### EGFR mutation analysis

Cytologic or histologic specimens were examined for *EGFR* mutations by the PNA-LNA PCR clamp method as reported previously [10].

### Fluorescence *in situ* hybridization (FISH)

**Figure 1:**
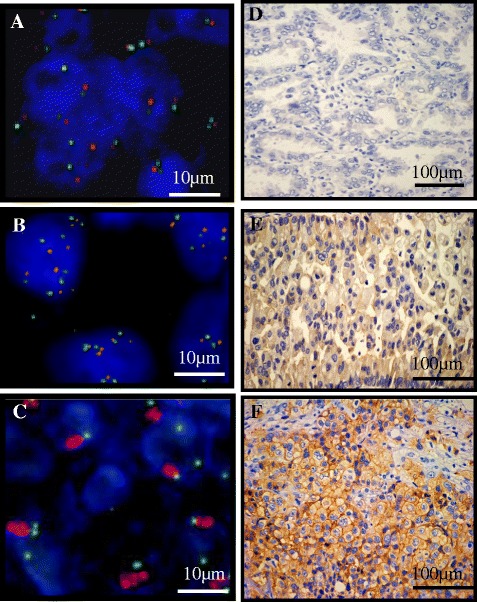
**FISH analysis of the** ***MET*** **gene and immunohistochemical staining for MET protein.** Gene copy numbers (GCNs) and amplification of the *MET* gene were examined by fluorescence *in situ* hybridization (FISH). **(A)** FISH negativity was defined as mean MET per cell < 5 copies. **(B)** High polysomy was defined as 5 copies≦mean MET per cell. **(C)** Amplification was defined as 2≦*MET gene* (red)/*CEP7q* (green) per cell. FISH positivity consisted of high polysomy and amplification. **(D)** Low level of MET protein expression in lung adenocarcinoma tissues. **(E)** Moderate level of MET protein expression in lung adenocarcinoma tissues. **(F)** High level of MET protein expression in lung adenocarcinoma tissues. Bars indicate 100 μm.

Gene copy numbers (GCNs) and amplification of *MET* gene were examined by FISH. The tissue sections were then hybridized with Met (TexRed)/CEN7q (FITC) Dual Color FISH Probe. (GSP Laboratory, LCI Medience Corporation, Chiba, Japan). The number of fluorescence signals was counted independently by two investigators using an Axio Vision microscope (Carl Zeiss, Oberkochen, Germany). *MET* GCN was determined by FISH with probes for Met (TexRed)/CEN7q (FITC) Dual Color FISH Probe. FISH positivity was estimated using the standard Colorado criteria (gene amplification; 2≦*MET gene* (red)/*CEP7q* (green) per cell plus high polysomy; 5 copies≦mean MET per cell) [9,11-13] (Figure 1 ).

### Immunohistochemical (IHC) analysis

For IHC of MET, formalin-fixed paraffin-embedded tissue sections were stained by the immunoperoxidase method with avidin-biotin complex as described previously [14]. The slides were incubated with a primary antibody against MET, clone SP44 (1:50, Spring Bioscience, Pleasanton, CA). Positive is defined as the membranous and/or cytoplasmic staining in greater than 10% tumor cells with moderate level and high level. Negative is defined as the membranous and/or cytoplasmic staining less than 10% tumor cells and the membranous and/or cytoplasmic staining in greater than 10% tumor cells with low level. [14] (Figure 1D-F).

### Statistical analyses

Correlations between response rate and clinical characteristics were compared by Fisher’s exact test. Progression-free survival (PFS) was calculated from the time of gefitinib therapy to time of disease progression or last disease assessment. Overall survival (OS) was calculated from the time of gefitinib therapy to patient death or last contact. Kaplan–Meier survival curves were drawn for PFS and OS and compared by log-rank test. Univariate and multivariate analyses were performed using the Cox regression model. Statistical significance was defined as p < 0.05 for each analysis. All statistical analyses were carried out using Stat Flex version 7 [11].

## Results

### Status of *EGFR* and *MET* genes before gefitinib therapy and at treatment failure

Eleven of 35 patients (31%) had *MET* gene activation as estimated by FISH analysis. High polysomy was observed in 10 patients (29%) and amplification was in 1 patient (3%) of 11 *MET* FISH-positive cases (Table 1) (Figure 1A-C). Nine cases were available for evaluation of *EGFR* and *MET* status by FISH at the failure of gefitinib therapy (Table 1). T790M secondary mutation was detected in 2 of 9 patients examined at gefitinib treatment failure (case**s** 3, 15). Exon 19 deletion was not detected at gefitinib failure in one case (case 2). Four cases were also observed to be *MET* FISH-positive at the failure of gefitinib treatment (case**s** 1, 2, 3, 7). One case with *MET* amplification before initiation of therapy was also found to harbor amplification during relapse (case 1). At gefitinib failure, coexistence of T790M and MET high polysomy of the *MET* gene were observed in one case (case 3). *MET* gene status was found to be not related to clinicopathological factors (Table 2).

**Table 2:** **Clinicopathologic characteristics of the 35 lung adenocarcinoma cases**

			***MET gene FISH status***				
			**FISH-negative**		**FISH-positive**		**p-value**
	**Total**	**%**	**Total**	**%**	**Total**	**%**	
**Variables**	35	100	24	100	11^a^	100	
**Age**							
<65	13	37	9	38	4	36	
65≦	22	63	15	63	7	64	1.00
**Gender**							
Male	15	43	12	50	3	27	
Female	20	57	12	50	8	73	0.28
**Smoking status**							
Current and former smoker	14	40	10	42	4	36	
Never smoker	21	60	14	58	7	63	1.00
**Stage**							
III	4	11	3	13	1	9	
IV + Relapse	31	89	21	87	10	91	1.00
***EGFR mutation subtype***							
Exon19 deletion	25	71	19	79	6	55	
*Exon21 L858R*	10	29	5	21	5	46	0.23
**Response to Gefitinib**							
CR + PR	31	89	22	92	9	82	
SD + PD	4	11	2	8	2	18	0.57

^a^High polysomy was observed in 10 patients (28.5%) and amplification was in 1 patients (2.9%) of 35 NSCLC patients.

### Relationship between GCNs and MET protein expression

Next, we assessed MET protein expression levels in 9 available tumor sections by IHC and evaluated the relationship between the expression of MET and GCNs (Table 1) (Figure 1D-F). One case with *MET* amplification showed high MET protein expression (case 1), and correspondingly, no positive MET staining was observed in the 4 patients without high polysomy (case**s** 15, 16, 17, 32). However, only one of four cases with high polysomy showed moderate level of MET expression (case 8). One limitation of our study was that the number of samples was small. Thus, analysis of MET protein expression by IHC could not confirm *MET* high polysomy status.

### Correlation between *EGFR* gene and gefitinib sensitivity

**Figure 2:**
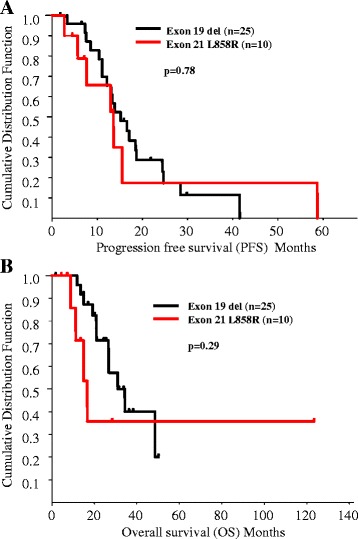
**The correlation between** ***EGFR*** **mutation type and prognosis in lung cancer. (A)** Kaplan-Meier estimates of progression-free survival of patients harboring exon 19 deletions (black) and L858R mutation (red) who had received gefitinib treatment. Median survival time of patients harboring exon 19 deletions (black) and L858R mutation (red) was 14.6 months and 13.1 months, respectively. The difference was not statistically significant (p = 0.78). **(B)** Kaplan-Meier estimates of overall survival of patients harboring exon 19 deletions (black) and L858R mutation (red) who had received gefitinib treatment. Median survival time of patients harboring exon 19 deletions (black) and L858*R* mutation (red) was 31.1 months and 15.3 months, respectively. The difference was not statistically significant (p = 0.29).

3000The *EGFR* gene mutation subtype was not found to be associated with sensitivity to gefitinib. Median PFS of patients harboring the L858R and exon 19 deletion mutations was 14.6 months and 13.1 months, respectively (p =0.78) (Figure 2A). Median OS of patients harboring the L858R and exon 19 deletion mutations was 15.3 months and 31.1 months, respectively (p = 0 .29) (Figure 2B).

### Correlation between *MET* gene status, EGFR-TKI sensitivity, PFS and OS after gefitinib treatment

**Figure 3:**
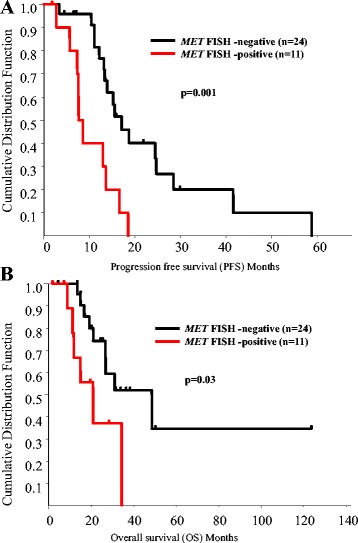
**Prognostic significance of** ***MET*** **FISH-positivity in lung cancer. (A)** Kaplan-Meier estimates of progression-free survival of FISH-negative (black) and -positive (red) patients who had received gefitinib treatment. Median survival time of FISH-negative (black) and -positive (red) patients was 15.9 months and 7.6 months, respectively. The difference was statistically significant (p = 0.001). **(B)** Kaplan-Meier estimates of overall survival of FISH-negative (black) and -positive (red) patients who had received gefitinib treatment. Median survival time of FISH-negative (black) and -positive (red) patients was 33.0 months and 16.8 months, respectively. The difference was statistically significant (p = 0.03).

Responses to gefitinib were not significantly different according to *MET* gene status (Table 2). However, PFS in *MET* FISH-positive patients was significantly shorter than in *MET* FISH-negative patients. Median PFS in *MET* FISH-negative and *MET* FISH-positive patients was 15.9 months and 7.6 months, respectively (p = 0.001) (Figure 3A). One case with *MET* gene amplification had PR with shorter PFS at 7.3 months (case 1) (Table 1). Furthermore, median OS in *MET* FISH-negative and *MET* FISH-positive patients was 33.0 months and 16.8 months, respectively (Figure 3B), and the difference in OS between these cases was statistically significant (p = 0.03). Univariate Cox regression analysis revealed that *MET* FISH-positive cases showed a significantly poorer outcome than negative cases (hazard ratio for progression and death in *MET* FISH-positive cases relative to *MET* FISH-negative cases, 3.83 (p = 0.008) and 2.25 (p = 0.03), respectively (Table 3).

**Table 3:** **Univariate Cox proportional hazards models of factors associated with death and progression for all cases**

**Hazard ratios for death in according to prognostic factors**				
**Characteristics**	**Comparison**	**Hazard ratio [95% CI** ^**a**^ **]**		***P value*** ^**b**^
**Age**	<65 yr vs. ≥65 yr	1.26	[0.50-3.17]	0.62
**Gender**	Female vs. Male	0.59	[0.23-1.49]	0.27
**Smoking status**	Never smoker vs. Ever smoker	0.41	[0.15-1.13]	0.08
**Stage**	III vs. IV/Relapse	1.46	[ 0.34-6.37]	0.61
***EGFR mutation type***	Exon19 deletion vs. Exon 21 L858R	1.90	[0.72-5.00]	0.62
**MET FISH**	Negative vs. Positive	2.25	[1.07-4.74]	**0.03**
**Hazard ratios for progression in according to prognostic factors**				
**Characteristics**	**Comparison**	**Hazard ratio [95% CI** ^**a**^ **]**		***P value*** ^**b**^
**Age**	<65 yr vs. ≥65 yr	0.52	[0.23-1.15]	0.11
**Gender**	Female vs. Male	0.88	[0.40-1.93]	0.75
**Smoking stutus**	Never smoker vs. Ever smoker	1.11	[0.51-2.42]	0.79
**Stage**	III vs. IV/Relapse	1.04	[0.30-3.64]	0.95
***EGFR mutation type***	Exon19 deletion vs. Exon 21 L858R	1.15	[0.45-2.91]	0.77
**MET FISH**	Negative vs. Positive	3.83	[1.75-8.38]	**0.0008**

^a^ *Abbreviation*: *CI* Confidence interval.

^b^Cox regression analysis. P values of < .05 are shown in bold.

## Discussion

In this study, we evaluated by FISH analyses, the *MET* status of 35 lung adenocarcinoma patients with *EGFR* gene mutations who received gefitinib therapy. *MET* gene amplification before EGFR-TKI therapy was observed by high-throughput FISH analysis [8]. This finding suggests that possibly a small population of cancer cells with *MET* gene amplification can become major clones after EGFR-TKI treatment and upon accumulation of secondary genetic alterations, such as the T790M mutation. In our analysis, MET FISH-positivity was also detected after continued exposure to gefitinib. Of course MET status is only one of several molecular mechanisms that account for resistance to TKIs, and was the only one we investigated in the 35 cases. Other mechanisms could lead to acquiring drug resistance. For example, it is possible that many more of the relapsed, non-tested, cases carried T790M mutations and that this was greater factor shortening the survival rate in the few MET positives cases.

A recent report demonstrated that lung adenocarcinoma cases with a co-existence of positive *MET* FISH status and EGFR mutation had shorter disease-free survival (DFS) as well as OS after resection [15]. However, the relationship between *MET* FISH status and clinical outcomes of treatment with EGFR-TKIs has not been evaluated in previous reports [15,16]. Our data showed that the response to gefitinib was not significantly different according to *MET* gene status. Nevertheless, *MET* FISH-positive patients revealed not only significantly shorter PFS but also OS from the beginning of gefitinib therapy as compared to *MET* FISH-negative patients. Previous reports demonstrated that gain of *MET* GCNs may be related to the elevation of MET protein expression and its phosphorylation [17]. In this study, FISH amplification patients had high MET protein expression. However, MET high polysomy status could not be verified by IHC. More samples will be needed for evaluating the correlation between MET GCNs and MET protein expression. These results suggest that detection of high polysomy and amplification of *MET* gene by FISH may be useful for predicting short PFS and OS after gefitinib treatment in Lung Adenocarcinoma with EGFR mutation.

Transgenic mouse models for lung cancer that express *EGFR* mutation with MET overexpression demonstrated that monotherapy targeting either EGFR or MET did not show tumor regression [17]. In contrast, combination therapies targeting both EGFR and MET simultaneously were significantly effective against EGFR TKI-resistant tumors with mutant *EGFR* and MET activation [18]. Demonstrating this, a recent phase II study showed that previously-treated NSCLC patients using a combination therapy of OAM4558g (MET-MAb) plus erlotinib versus just erlotinib alone reported that the MET-MAb plus erlotinib therapy significantly improved PFS and OS, resulting in a near 3-fold reduction in the risk of death. This benefit was observed in patients with MET activation, as evaluated by IHC [19]. However in the Phase III trials, when those patients selected for high MET expression were treated OAM4558g added to erlotinib, it was shown to be not superior to erlotinib alone [20]. In light of this, analyses of EGFR-TKI failing with EGFR mutant cases will be required.

Yet another phase II study with combination therapy in previously-treated NSCLC patients, this one with erlotinib plus tivantinib (ARQ 197) (MET-TKI) versus just erlotinib alone in previously-treated NSCLC patients showed that the median PFS was longer in the erlotinib plus tivantinib group than in the erlotinib alone group, particularly among patients with *KRAS* mutations, although this study did not meet its primary end point [21]. Based on these phase II trials, additional phase II trials of erlotinib plus tivantinib for EGFR-mutated NSCLC patients after failure of EGFR-TKI treatment are now ongoing in Asian countries. A MET inhibitor combined with EGFR-TKI may be effective in *MET* FISH-positive patients with *EGFR* mutations.

## Conclusions

Pre-gefitinib MET FISH status may be useful for predicting PFS and OS after Gefitinib treatment in lung adenocarcinoma with EGFR mutation and for selecting the patients who would benefit from EGFR-TKI and MET inhibitor therapy. Correlations between *MET* gene status and clinical outcome for EGFR-TKI should be further evaluated using larger scale samples.
